# Castleman Disease: A Rare Condition with Endocrine Manifestations

**DOI:** 10.7759/cureus.380

**Published:** 2015-11-17

**Authors:** Carmen E Cervantes, Ricardo Correa

**Affiliations:** 1 Laboratory of Biochemistry and Genetics, NIDDK/NIH; 2 Medicine Faculty, Universidad Autonoma de Guadalajara; 3 Endocrinology, NICHD/NIH

**Keywords:** castleman disease, angiofollicular lymph node hyperplasia, poems syndrome, osteosclerotic myeloma

## Abstract

Castleman disease (CD) most commonly affects lymphoid tissues in the thorax, abdomen, pelvis, and neck. Extralymphatic tissues, such as lacrimal glands, lung, pancreas, larynx, parotid, meninges, and even muscles, have also been reported as sites. The etiology is unknown and its incidence has not been reported in the literature. Castleman disease can be classified clinically into a unicentric or multicentric form, depending on the number of lymph nodes involved, and histologically into a hyaline vascular variant, plasma cell, mixed cellular, or plasmablastic variant. The disease has a predominantly inflammatory background, reflected in high levels of vascular endothelial growth factor (VEGF) and interleukin-6 (IL-6). The role of cytokines in CD explains the clinical presentation. The clinical scenario varies widely, based mainly on the histologic type. Unicentric CD usually presents without symptomatology, whereas multicentric manifests with fatigue, abdominal or thoracic pain, cytopenias, and/or B- symptoms (10% weight loss in the last six months, nocturnal diaphoresis, and fever). The endocrinopathy has a wide range of manifestations, affecting either the pituitary or other target organs. Achieving the diagnosis is complicated and there is no laboratory or imaging pathognomonic for this disease. The gold standard is an excisional biopsy from an affected lymph node. The treatment depends on the type of CD. Unicentric CD has a good response to excisional surgery. However, in multicentric CD (MCD), surgery may provide transient relief of symptoms but with a rebound effect, so it is not considered a good method. The use of chemotherapy, monoclonal antibodies, glucocorticoids, and thalidomide has shown some improvement in MCD.

## Introduction and background

In 1956, Castleman disease (CD), also known as angiofollicular lymph node and giant lymph node hyperplasia, was first described in 13 subjects with mediastinal lymphadenopathy [1-4]. The most common affected sites include lymphoid tissues in the thorax in 70% of cases, abdomen and pelvis in 15%, and the neck in 15% [4-6]. Extralymphatic involvement may include lacrimal glands, lung, pancreas, larynx, parotid, meninges, and even muscles of the extremities [4, 6-12].

The etiology is unknown, although it has been associated with human herpesvirus-8 (HHV-8) infection, particularly, but not only, in HIV-positive patients [2]. CD has a clinical and histological classification described in Table 1 [2, 4].

**Table 1:** Castleman Disease Classification

Clinical (Depending on the Number of Lymph Nodes Involved)	Histological
Unicentric (UCD) (47%-81%)	Hyaline vascular
Multicentric (MCD)	Plasma cell
	Mixed cellular
	Plasmablastic variant (also known as HHV-8 associated MCD)

Systemic symptoms are mostly related to the presence of plasma cells, although some hyaline vascular form cases have displayed them as well [2]. Other clinical entities may accompany CD, such as lymphoid neoplasms, POEMS syndrome (coined to refer to polyneuropathy, organomegaly, endocrinopathy, M protein, and skin changes), amyloidosis, and osteosclerotic myeloma. The diversity of its presentation stands as a challenge for its diagnosis [2, 11].

## Review

### Epidemiology

Due to the rarity of the disease, there is no incidence reported in the literature. However, a recent study attempted to calculate it, obtaining an incidence of 21 and 25 per million person-years, which confirms its orphan disease status. Likewise, they estimated that 23% of the patients had multicentric Castleman disease (MCD) [13]. The syndrome appears to be more common among Asian people, especially Japanese [3, 8, 13].

The hyaline vascular type usually presents as unicentric Castleman disease (UCD) in 76% to 91% of cases, involving a single node or a localized group of nodes. It affects both genders equally. The median age reported lies in the fourth decade [2, 13]. The plasma cell variant is regularly multicentric, although it occurs in 9% to 24% of localized UCD (and shares its epidemiologic characteristics). Most plasma cell variants in MCD affects subjects in the sixth decade [2].

In HIV-positive individuals, MCD tends to occur in the fourth decade of life, prevailing in men [2, 13].

### Physiopathology

The disease has a predominant inflammatory background, reflected in high levels of vascular endothelial growth factor (VEGF), c-Myc-dependent transcription factor and IL-6. This cytokine acts both as a differentiation factor for macrophages as well as a hepatocyte and B cell stimulating factor. It also has a role in the modulation of immune and central nervous systems and bone metabolism [14-15]. Likewise, a viral IL-6 (analog to its human form) encoded by HHV-8 activates the Janus kinase/signal transducer and activator of transcription (JAK-STAT) signaling pathway, which stimulates more cell types and potentiates the effect of human IL-6. This is translated into an increased angiogenesis and proliferation of polyclonal IgM-lambda plasma cells in the follicles of affected nodes [2, 4, 14]. Autoantibodies produced by plasma cells have been suggested as the effectors of the endocrinopathy; nonetheless, no circulating forms against the hormones or its receptors have been found [14, 16]. Apparently, VEGF affects the endocrine axes by inducing an imbalance in angiogenic factors that regulate hormonal secretion [15].

IL-6 causes a systemic response by stimulating hepatocytes to produce C-reactive protein [4, 14]. It also increases the levels of the hormone hepcidin, which plays a leading role in anemia of chronic diseases [17-18].

Bone remodeling depends on cytokine stimulation of resorption and apposition, featuring RANKL (receptor activator of nuclear factor kappa-B ligand) as the predominant effector. However, IL-6 also exhibits an important function, whose relevance rises in this disease as its blood levels reach upper limits. IL-6 receptors are expressed in low levels in osteoblasts, enhancing their differentiation into osteocytes and inhibiting apoptosis. Moreover, in an indirect way, IL-6 displays an inhibitory effect on osteoclasts, thus, decreasing bone resorption [14].

The roles of both cytokines (IL-6 and VEGF) in CD have explained interchangeably the clinic presentation of these patients. An elevated VEGF level has been considered one of the criteria for POEMS syndrome, and due to the higher prevalence of bone lesions in this group, its levels have been associated with the degree of bone compromise [14, 16].

Multicentric and HHV-8 positive variants are risk factors for the development of complications, such as Hodgkin and non-Hodgkin lymphomas, as well as Kaposi sarcoma [2, 13].

### Clinical manifestations

The clinical scenario varies widely, based mainly in its histologic type. UCD (usually the hyaline vascular variant) can be asymptomatic or present a course with manifestations secondary to compression. However, detailed cases have a course with systemic signs [19-20]. On the other hand, MCD has shown a greater clinical spectrum in connection with the presence of plasma cells, which ranges from fatigue and B-symptoms to pleural effusions and organomegaly that may mimic systemic infections or even hematologic neoplasms  [4].

Cronin, et al. established two subgroups of MCD, though not well defined because they were not distinguished in older reports: MCD associated with HHV-8 and MCD not otherwise specified [2]. The difference between both groups is relevant since MCD associated with HHV-8 is usually present in HIV patients; apparently, only a single case has been reported of MCD involving an HIV-positive patient with a negative HHV-8 viral panel [2, 20]. This subgroup has a different course and a poorer prognosis, as it tends to progress to a large B-cell lymphoma (HHV-8-positive plasmablastic lymphoma) [2].

Systemic manifestations, such as fatigue, abdominal or thoracic pain, cytopenias, and B-symptoms, are most common in MCD [4, 21-22].

POEMS syndrome accounts for polyneuropathy, organomegaly, endocrinopathy, M protein or gammopathy, and skin changes. Its diagnosis is made with two major criteria and one minor criterion. The major criteria comprise polyneuropathy, monoclonal peak, Castleman disease, sclerotic bone disease, and elevated VEGF. The minor criteria consist of organomegaly, signs of extravascular volume overload (pleural effusion, ascites, and edema), endocrinopathy, polycythemia, papilledema, and skin changes. Organomegaly stands for lymphadenopathy, hepatomegaly, or splenomegaly [16, 21].

Polyneuropathy starts as a distal sensory loss, which later includes motor disabilities. Nerves have segmental demyelination, axonal degeneration, and immunostaining that show anti-myelin antibodies [21].

Endocrinopathy has a wide range of manifestations, affecting either the pituitary or target organs. Secondary hypogonadism stands as the most common endocrine dysfunction in 70% of cases. Reports from the Mayo series, only available for males, features erectile dysfunction as the predominant symptom, followed by gynecomastia [16, 21]. The second most common endocrine manifestation is hypothyroidism in 58% of the patients, although it has a mild course. In third place, the Mayo series reported 48% with either impaired glucose tolerance or diabetes. The adrenal is rarely affected, but when it is, it usually manifests as primary adrenal insufficiency. Though prolactin is mildly elevated in most patients, only a small group exhibits irregular menses or galactorrhea. Parathyroids are rarely assessed, but some cases with secondary hyperparathyroidism have been described. Therefore, it is important to establish if calcium levels are altered due to these glands' dysfunction or secondary to bone turnover [16, 21, 23]. The pituitary compromise is infrequent, with few reports of adenomas resulting in panhypopituitarism and acromegaly [21, 24-25].

In 50-90% of patients, skin changes have been described. Hyperpigmentation is the most common sign while others include skin thickening, clubbing, white nails, acrocyanosis, and hirsutism [16, 21]. Paraneoplastic pemphigus has been linked to CD and recognized as a bad prognosis factor, although mostly associated with CD itself and not as part of the POEMS syndrome [26-28].

Sclerotic bone lesions are well described, mostly in association with POEMS syndrome, although isolated reports of MCD with a bone compromise without the POEMS spectrum are also available. In most cases, they are asymptomatic and are identified when an FDG-enhancing PET scan is performed to evaluate the extent of the disease. When biopsied, bone marrow has normal cellularity or a slight increase in plasma cells [29].

### Diagnosis

Laboratory studies can show anemia, thrombocytosis, or thrombocytopenia. C-reactive protein, IL-6, and VEGF should be measured as well as liver function tests [22, 26]. One should note that, despite the systemic manifestations, some published cases have described normal levels of these cytokines and a normal CBC [4, 14, 22].

The endocrine workup must include central hormones and those produced in target organs. It should be performed not only initially, but also as a follow-up due to the emergence of new endocrinopathies in patients with POEMS syndrome [21].

There are no radiologic criteria; in general, MRI and CT may help identify hypervascularity by showing a higher enhancement in the hyaline vascular variant [30]. Calcifications can be found in this form but are rare in the plasma cell type, which usually shows lower enhancement [31].

Classic UCD appears as a solitary enlarged lymph node or clusters of nodes with intense homogeneous contrast enhancement. In fewer cases, imaging shows a dominant mass surrounded by normal lymphadenopathy (40%) or matted lymphadenopathy alone without a dominant mass (10%) [4, 31]. Nonetheless, depending on the location, UCD can mimic a variety of diseases, from thymoma and neural–crest-derived neoplasms in the mediastinum to pancreatic cancer and carcinoid in the abdomen [4].

MCD frequently exhibits generalized lymphadenopathy, comprising the abdomen, pelvis, mediastinum, neck, axilla, and inguinal regions. However, these nodal lesions resemble reactive or neoplastic diseases. Pulmonary centrilobular nodular parenchymal opacities suggest lymphocytic interstitial pneumonitis, common in MCD [4, 11]. 

The immunocompromised groups of patients with HHV8-CD pose a higher degree of diagnostic difficulty, as lesions closely resemble opportunistic infections and lymphoma [4].

A positron emission tomography (PET) scan may be performed as follow-up and to stage the disease [4]. Usually, sclerotic bone lesions are a diagnostic finding. They appear as focal areas of more intense FDG uptake in the bone [29, 31].

The gold standard for diagnosis is an excisional biopsy from the lymph node; the fine needle aspiration is commonly undiagnostic. The hyaline vascular variant will show normal follicles with larger mantle zones forming concentric rings around atretic germinal centers, known as the "onion-skin phenomenon" [4]. Germinal centers exhibit increased overall vascularity, although there is a prominent penetrating vessel. The sinusoidal vessels are also enlarged, and perivascular hyalinization has been described [2, 4]. 

The plasma cell type histology is not unique and can be confounded with other inflammatory and infectious diseases. The lymph node architecture is preserved; germinal centers show mild hyperplasia with expanded mantle zones and paracortical plasmacytosis [2]. Abnormal clusters of monotypic plasma cells with restricted lambda IgG or IgA light chains have been reported as well [4].

HHV-8-associated CD shares plasma cell variant characteristics but is ruled out by clinical data and immunohistochemical staining [4].

### Treatment

UCD has a good response to excisional surgery or localized radiation if that is not feasible. A complete surgical resection is the gold standard because of its excellent prognosis without remission after 20 years of follow-up [4, 32-35]. Intensity-modulated radiation therapy (IMRT) has been described as a novel radiotherapeutic approach for diseases located mainly in the thorax that represent difficult surgical access. It is better than 3D conformal techniques, as it reduces the dose gradient and toxicity to the surrounding normal tissues [36].

Due to the several systemic manifestations of MCD, surgery may give transient relief of symptoms but with a rebound effect, so it is not considered a good method [34-35, 37].

Among the drugs described, glucocorticoids (usually used as adjuvant therapy) improve symptoms and correct blood analyses abnormalities. However, the benefit is temporary. Antiangiogenic drugs, such as thalidomide, have also produced improvement [32, 38].

Currently, multidrug schema chemotherapy stands as the best option. It has induced remission in 50% of cases with CHOP (cyclophosphamide, hydroxydaunorubicin (doxorubicin), oncovin (vincristine), and prednisone) or in 67% with CVAD (cyclophosphamide, vincristine, Adriamycin, and dexamethasone) [32-33, 38].

Monoclonal antibodies, such as rituximab (anti-CD20) and tocilizumab (anti IL-6 receptor), have been described without enough evidence. However, one recently anti IL-6 drug, siltuximab, has shown efficacy and safety, reason by which FDA has already approved it as treatment [39-40].

Antiviral agents, anti HHV-8, such as ganciclovir, valganciclovir, cidofovir, or foscarnet, play a theoretical role. Nonetheless, a good response of valganciclovir and CHOP chemotherapy has been described in HIV-positive patients [32, 38]. This therapy must go in conjunction with highly active anti-retroviral therapy (HAART) after CD is diagnosed in this group [20].

Despite the therapy, the prognosis of MCD is poor, although it depends on the progression rate, infections, and comorbidities. These patients usually die from sepsis, multi-organ system failure (due to systemic inflammation), or malignancy (lymphoma is the most frequent) [38].

A radiological follow-up with CT is suggested six to 12 months after therapy for UCD. Subjects with MCD need a radiologic and serum follow-up with a complete blood count, liver function test, and C-reactive protein every six months or when there is a suspected clinical recurrence [4, 38].

## Conclusions

Castleman disease is a rare condition of unknown etiology that affects lymphoid tissues in diverse locations. The clinical scenario depends mostly on the histologic type, which includes plasma cell, mixed cellular, plasmablastic, and hyaline vascular variant, the latter being the most common. The clinical presentation is explained through a number of cytokines (particularly IL-6 and VEGF) produced mainly by those variants with plasma cells. Among them, we find fatigue, pain in different locations of the body, cytopenias, and B- symptoms. The endocrinopathy is manifested as part of POEMS syndrome, where the pituitary gland is the most commonly affected organ. Excisional surgery stands as an excellent therapy for unicentric Castleman disease, while chemotherapeutic schemas with CHOP (cyclophosphamide, doxorubicin, vincristine, and prednisone) or with CVAD (plus dexamethasone) are better options for multicentric CD cases. The wide spectrum of clinical manifestations makes the diagnosis a challenge. This is one of the reasons that the incidence and prevalence of the disease are not only underestimated but has not even been well-established. Therefore, it is important that physicians consider this clinical entity so patients can receive adequate and early treatment.
